# *De novo* assembly and characterisation of the field pea transcriptome using RNA-Seq

**DOI:** 10.1186/s12864-015-1815-7

**Published:** 2015-08-16

**Authors:** Shimna Sudheesh, Timothy I. Sawbridge, Noel OI Cogan, Peter Kennedy, John W. Forster, Sukhjiwan Kaur

**Affiliations:** Department of Economic Development, Jobs, Transport and Resources, Biosciences Research Division, AgriBio, Centre for AgriBioscience, 5 Ring Road, Bundoora, VIC 3083 Australia; Department of Economic Development, Jobs, Transport and Resources, Biosciences Research Division, Grains Innovation Park, Horsham, VIC 3401 Australia; School of Applied Systems Biology, La Trobe University, Bundoora, VIC 3086 Australia

**Keywords:** Grain legume, Second-generation DNA sequencing, *De novo* assembly, Sequence annotation, Tissue-specific gene expression, Plant breeding

## Abstract

**Background:**

Field pea (*Pisum sativum* L.) is a cool-season grain legume that is cultivated world-wide for both human consumption and stock-feed purposes. Enhancement of genetic and genomic resources for field pea will permit improved understanding of the control of traits relevant to crop productivity and quality. Advances in second-generation sequencing and associated bioinformatics analysis now provide unprecedented opportunities for the development of such resources. The objective of this study was to perform transcriptome sequencing and characterisation from two genotypes of field pea that differ in terms of seed and plant morphological characteristics.

**Results:**

Transcriptome sequencing was performed with RNA templates from multiple tissues of the field pea genotypes Kaspa and Parafield. Tissue samples were collected at various growth stages, and a total of 23 cDNA libraries were sequenced using Illumina high-throughput sequencing platforms. A total of 407 and 352 million paired-end reads from the Kaspa and Parafield transcriptomes, respectively were assembled into 129,282 and 149,272 contigs, which were filtered on the basis of known gene annotations, presence of open reading frames (ORFs), reciprocal matches and degree of coverage. Totals of 126,335 contigs from Kaspa and 145,730 from Parafield were subsequently selected as the reference set. Reciprocal sequence analysis revealed that c. 87 % of contigs were expressed in both cultivars, while a small proportion were unique to each genotype. Reads from different libraries were aligned to the genotype-specific assemblies in order to identify and characterise expression of contigs on a tissue-specific basis, of which 87 % were expressed in more than one tissue, while others showed distinct expression patterns in specific tissues, providing unique transcriptome signatures.

**Conclusion:**

This study provided a comprehensive assembled and annotated transcriptome set for field pea that can be used for development of genetic markers, in order to assess genetic diversity, construct linkage maps, perform trait-dissection and implement whole-genome selection strategies in varietal improvement programs, as well to identify target genes for genetic modification approaches on the basis of annotation and expression analysis. In addition, the reference field pea transcriptome will prove highly valuable for comparative genomics studies and construction of a finalised genome sequence.

**Electronic supplementary material:**

The online version of this article (doi:10.1186/s12864-015-1815-7) contains supplementary material, which is available to authorized users.

## Background

Field pea is a member of the Galegoid clade of the Papilionoideae sub-family of the Fabaceae family, and is a cool-season grain legume which is cultivated world-wide (6.4 million hectares per year) for both human consumption and stock-feed purposes [1]. Pea is a self-pollinated diploid species (2n = 2× = 14) with a genome size of c. 4,300 Mb, which is approximately 10-fold larger than that of the most closely related model legume species, *Medicago truncatula* Gaertn. (c. 500 Mb). This expansion is largely due to a substantial quantity of repetitive DNA (c. 50-70 % of the nuclear genome complement) composed of various families of mobile genetic elements [2, 3]. As a consequence, the exomic component (gene space) of the pea genome constitutes a much lower proportion of total genomic DNA than for legume species such as *M. truncatula* [4], *Lotus japonicus* L. (c. 472 Mb) [5]*,* and chickpea (*Cicer arietinum* L.) (c. 740 Mb) [6], which have been the subjects of whole genome sequencing activities.

Enrichment of genetic resources for field pea is essential in order to provide effective tools for molecular breeding, with the aim of improving both productivity and quality of the crop, and sustainability of farming practices. Genic regions, which provide the primary targets for such activities, may be obtained by direct sampling of genomic sequences. However, given the relatively low proportion of such regions within the field pea genome, a more attractive current option is indirect sampling through access to the transcriptome, the latter being the actively transcribed sub-component of the genome in a given cell type at any particular stage of the life-cycle.

The increasingly high-throughput nature and declining costs of second-generation DNA sequencing have provided a durable solution for transcriptome analysis based on direct sequence evaluation through transcript discovery, identification of the transcriptional structure of a gene, detection of alternate splicing patterns, and quantification of expression levels [7, 8]. RNA sequencing (RNA-Seq), has been demonstrated to be superior to earlier methods such as microarrays for detection of low abundance transcripts, differentiation of biologically critical isoforms and identification of genetic variants such as alternative alleles [9]. Extensive transcript expression profiling has previously been performed in order to provide insight into the roles of different functional developmental modules (for *Arabidopsis thaliana*) [10], or different cell types and developmental processes (*Oryza sativa*) [11]. For legumes, specific transcriptional activities of genes across tissues and/or between organs such as nodule [12], seed [13] and flower [14], and their respective developmental stages, have been identified. In the recent past, RNA-Seq has been performed for genome-wide transcriptome characterisation of both model and non-model plant species including maize (*Zea mays*) [15, 16], perennial ryegrass (*Lolium perenne* L.) [17] and soybean (*Glycine max* [L.] Merr.) [18]. RNA-Seq has also been employed to understand transcriptomic dynamics during plant responses to different biotic [19, 20] and abiotic stresses [21, 22].

Previous transcriptome analysis studies of field pea have been performed using second-generation DNA sequencing technologies, specifically the Roche 454 pyrosequencing system, mainly for use in the development of genetic markers [23–25]. Considerable progress has been made in the development of pair-cross specific [25–28] and consensus genetic linkage maps [25, 27, 28], based on use of such markers. Advances in genomics technologies now provide opportunities to develop substantially enriched genomic resources for field pea in order to assist accelerated delivery of improved cultivars. As the genome sequences of a number of legume species are now available, including *M. truncatula* [4], *L. japonicus* [5], chickpea [6], soybean [29] and pigeonpea (*Cajanus cajan* [L.] Millsp.) [30], corresponding data may be exploited for interpretation of transcriptome resources from less-well characterised taxa, such as field pea, to support gene annotation and comparative genome analysis.

In the present study, comprehensive transcriptome sets were generated from two genotypes of field pea that differ in terms of seed and plant morphological characteristics through use of RNA-Seq, followed by assembly, comparison to gene complements in related species, sequence annotation and assessment of tissue-specific expression. The resulting data provides a large-scale resource for the development of tools for molecular breeding of this important grain legume species.

## Methods

### Plant materials

Six plants from each of the two field pea cultivars, Kaspa (semi-leafless variety with medium height and produces spherical medium sized dun-type grain) and Parafield (conventional plant morphology and produces large sized dun-type grain) were maintained in glasshouses at 22 ± 2 °C under a 16/8-h (light/dark) photoperiod in individual pots filled with standard potting mix at the premises of DEDJTR - Bundoora, Victoria, Australia. Leaf, stipule, stem, tendril tissues from multiple nodes (at different developmental stages) as well as the root and root-tip tissues were collected from 4 weeks-old plants (three replicates per genotype). Fully open flowers, stamens, pistils, immature pods (10–14 days after flowering), immature seeds (20–25 days after flowering) and nodules (from 3 months old plants) were collected from three replicates of the two genotypes. To collect seedlings (7 days old), Kaspa and Parafield seeds (three replicates) were germinated on moist Whatman filter paper in falcon tubes and maintained in growth chambers at 22 ± 2 °C under a 16/8-h (light/dark) photoperiodic regime. After harvest, tissues were frozen immediately in liquid nitrogen and stored at −80 °C until required. For RNA extraction, replicates from individual tissues were pooled in equal proportion (by weight).

Total RNA was extracted using the RNeasy® Plant Mini Kit (QIAGEN, Hilden, Germany) following manufacturer’s instructions. A slight modification of the procedure was performed in order to extract RNA from immature seeds, which involved the addition of polyvinylpyrrolidone (PVP-40) (Sigma-Aldrich, Missouri, USA) to 450 μl of Buffer RLT containing 10 μl/ml β-mercaptoethanol (Sigma-Aldrich) to a final proportion of 2 % (w/v), and the remainder of the protocol was implemented according to manufacturer’s instructions. Aliquots of purified RNA were stored at −80 °C. The concentration of RNA was confirmed using a spectrophotometer (Thermo-Scientific, Delaware, USA) at the two wavelength ratios of A260/230 and A260/280 nm. The integrity of total RNA was determined by electrophoretic separation on 1.2 % (w/v) denaturing agarose gels.

### Library preparation

The polyA-containing (mRNA) fraction was isolated from total RNA (1 μg) using Dynabeads® with oligo (dT)_25_ residues covalently coupled to the surface (Life Technologies Australia Pty Ltd, Victoria, Australia). After purification, mRNA was fragmented by random shearing using heat treatment in the presence of Mg^2+^ ions. The resulting small fragments were used as templates to synthesise first-strand cDNA using random hexamer priming and the Bioline Tetro cDNA Synthesis Kit (Bioline US Inc., Massachusetts, USA). Second strand synthesis was performed in a solution containing NEBuffer 2 (New England Biolabs Ltd., Hitchin, United Kingdom), dNTPs (Bioline US Inc.), RNaseH (New England Biolabs) and DNA polymerase I (Thermo-Scientific). Subsequently, the ends of nascent cDNAs were polished by the addition of T4 DNA Polymerase (New England Biolabs) and Klenow DNA Polymerase (New England Biolabs) followed by paired-end adapter ligation to both termini of the cDNA fragments. Templates were then subjected to amplification using Phusion (Thermo-Scientific) DNA polymerase. The amplified libraries were pooled in equimolar amount and assessed by loading of a 1 μl aliquot on an Agilent Bioanalyzer 1000 DNA chip according to the manufacturer’s instructions. Library quantification was performed using the KAPA library quantification kit (KAPA Biosystems, Boston, USA). All reads were pair-end sequenced using the HiSeq 2000 and MiSeq platforms (Illumina Inc., San Diego, USA).

### De novo *transcriptome sequence assembly*

After removal of adaptor sequences along with low quality reads, sequence reads from each cultivar were *de novo* assembled using two transcriptome assemblers, Trinity (Trinityrnaseq_r20131110) [31] with the default settings including a fixed *k*-mer size of 25 and SOAPdenovo-Trans v1.03 [32], with different *k*-mer sizes (35, 45 and 55). To evaluate the quality of the assemblies, N50 statistics, contig counts and contig length distributions were assessed. The assemblies of the two cultivars were subsequently labelled as being derived from Kaspa and Parafield, respectively. Contigs from the assembly were further combined using CAP3 assembler with 95 % identity and minimum of 50 bp overlap to produce longer, more complete consensus sequences [33]. The CAP3 software removed the redundancies generated within assembly by consolidating the transcripts using overlap-layout-consensus (OLC) approach [33].

### Functional annotation and classification of the transcriptome

**Fig. 1:**
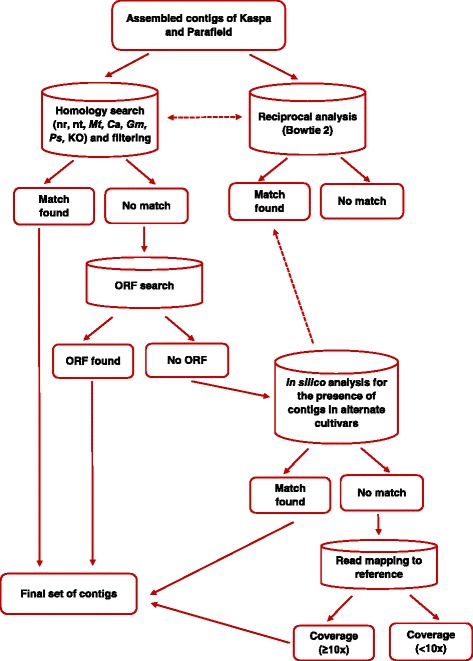
Computational pipeline for the functional annotation and classification of the field pea transcriptome

A workflow detailing the process of annotation and classification of the field pea transcriptome is shown in Fig. 1. All assembled contigs were searched against the non-redundant (nr) protein database maintained by NCBI using BLASTX [34] under the threshold parameter of E-value < 10^−10^. Any contigs that showed significant matches to non-plant databases were excluded from further analysis. For further assembly annotation, the contigs were utilised for similarity searches against the NCBI nucleotide (nt) database, genomes and coding DNA sequences (CDS) of *M. truncatula* (medicago v3.5) [4], chickpea [35] and soybean [36] using BLASTN with a threshold E-value of < 10^−10^ to capture any genomic sequences that may have been missed by BLASTX analysis. In order to obtain the final transcriptome set, results from the nr database were preferentially selected followed by those from the nt and other legume databases, where necessary. Both Kaspa and Parafield contigs were queried by BLASTN analysis against the pea chloroplast genome sequence (NCBI RefSeq NC_014057.1) in order to identify chloroplast-derived sequences.

Reads from each cultivar were also reciprocally compared to the assembly from the alternate cultivar as a reference using Bowtie 2 [37], in order to obtain estimates of common genic content between the two cultivars. The read counts of contigs that had no significant hit to the reference (other field pea genotype) were also examined, as these may represent contigs that were not assembled or expressed in the other transcriptome.

Subsequently, any contigs that were not annotated in the above mentioned procedures were searched for the presence of open reading frames (ORFs) using the ‘getorf’ function in the EMBOSS package [38] with minimum nucleotide size of 100 between START and STOP codons.

All annotated contigs were compared to the Kyoto Encyclopedia of Genes and Genomes (KEGG) database based on BLASTX queries. The KEGG pathway annotation was performed in the KEGG Automatic Annotation Server (KAAS) [39, 40] to further characterise the assembly.

To validate the current assembly, unigene sequences from previous pea transcriptome sequencing studies [23–25, 41] were aligned to the transcriptome dataset generated in the current study using BLASTN with an E-value < 10^−10^.

### Tissue-specific expression analysis

The trimmed reads from each library were aligned to the genotype-specific transcriptome through the use of Bowtie 2 [37], to obtain tissue-specific gene expression data. Relative expression based on read counts was used for this purpose, as the individual libraries varied in terms of read numbers. Normalisation of read counts from individual libraries of each cultivars were performed in MS Excel, based on the 75^th^ percentile value. For this, read counts from each libraries were multiplied by library specific scaling factor. This factor was calculated by dividing the maximum 75^th^ percentile value among different libraries by 75^th^ percentile value of the particular library (read normalisation = read counts × (the maximum 75^th^ percentile value/75^th^ percentile value of particular library). Previously, experiments were conducted comparing the reads normalisation as described above using MS Excel and RPKMs in “R Software”, revealing a 99 % correlation between the two methodologies (unpublished data). Read counts from different tissues were grouped into three clusters: reproductive tissues (flower, immature pod and immature seed); subterranean tissues (root, root-tip and nodule); and vegetative tissues (leaf, stem, stipule, seedling and tendril). The transcript expression profile was analysed in each instance.

The trimmed reads from root, root-tip and nodule libraries were further BLASTN analysed against various fungi and bacterial sequence (downloaded from NCBI) collections in order to estimate the possible presence of bacterial genes present within these tissues.

## Results

### De novo *sequence assembly of field pea transcriptome*

**Fig. 2:**
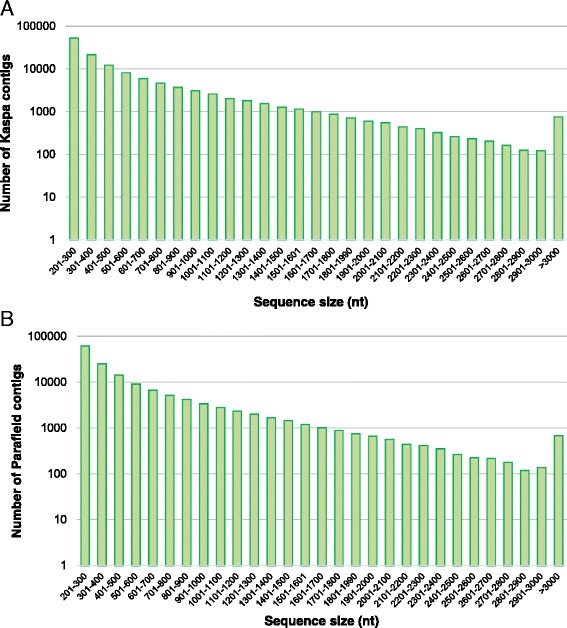
Length distribution of contigs from the (**a**) Kaspa-specific assembly, and (**b**) Parafield-specific assembly

To generate a comprehensive transcriptome dataset for field pea, a total of 23 cDNA libraries were generated from the various target tissues of the two cultivars, and were sequenced using both the HiSeq 2000 and MiSeq platforms. For cv. Kaspa, a total of 432 million paired-end reads with an average read length of 100 bp were obtained from the HiSeq 2000, as compared to 4 million paired-end reads with average read length of 250 bp from the MiSeq. The comparable figures from cv. Parafield were 372 million paired end reads with an average read length of 100 bp from the HiSeq 2000, and 3.7 million paired end reads with an average read length of 250 bp from the MiSeq. Details of the sequencing outcomes for each tissue-specific library of both varieties are provided in Additional file 1. An average of 35.2 million reads were generated per tissue type. After strict quality filtering, 408 million and 352 million reads (Table 1) from Kaspa and Parafield, respectively, were used for *de novo* assembly. Trinity assemblies were selected for further analysis, which produced 201,317 transcripts with N50 of 781 bp (Kaspa) and 226,701 transcripts with N50 of 772 bp (Parafield) (Table 2). Further CAP3 assembly in the former resulted in 129,282 contigs, while the latter constituted 149,272 contigs (Table 2). The contig length distribution from both assemblies is shown in Fig. 2.

**Table 1:** Details of the reads used for *de novo* transcriptome assembly

Cultivar	Source tissue	Number of reads used for assembly
Kaspa	Flower	49,261,131
	Immature pod	47,420,170
	Immature seed	33,879,162
	Nodule	31,101,358
	Pistil	25,767,536
	Root	33,938,588
	Root-tip	34,409,507
	Seedling	33,974,057
	Stamen	19,487,248
	Stem	35,122,274
	Stipule	33,995,906
	Tendril	29,401,978
	Total	407,758,914
Parafield	Flower	47,432,143
	Immature pod	18,675,349
	Immature seed	13,481,117
	Leaf	21,943,059
	Nodule	20,744,191
	Root	27,353,394
	Root-tip	27,865,916
	Seedling	38,346,614
	Stem	41,295,663
	Stipule	65,339,912
	Tendril	29,495,107
	Total	351,972,464

**Table 2:** Overview of sequencing outputs and assembly

	Kaspa	Parafield
Total raw reads	436,282,428	374,354,188
Total clean reads	407,758,914	351,972,464
Trinity		
Total number of contigs	201,317	226,701
N50	781	772
CAP3		
Total number of contigs	129,282	149,272
N50	757	717

### Functional annotation and classification of field pea transcriptome

**Fig. 3:**
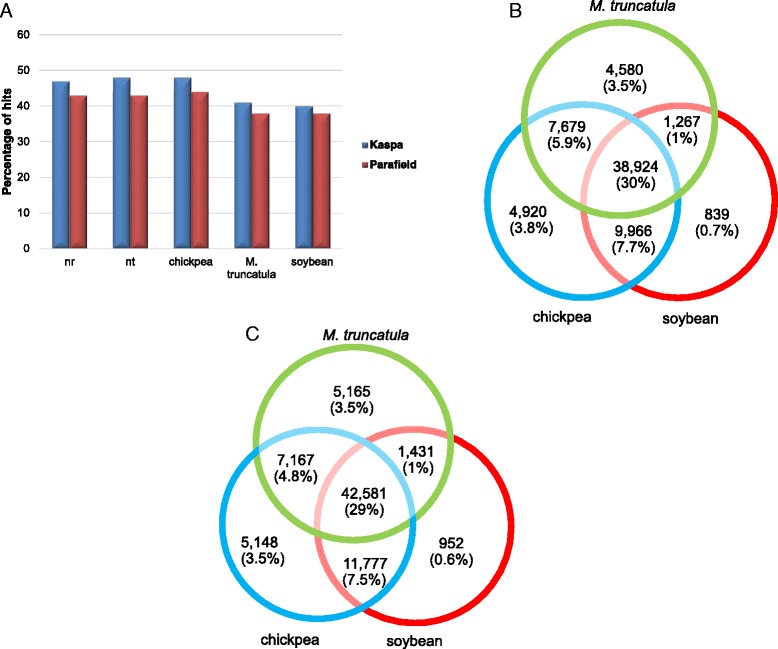
Sequence conservation of field pea contigs in comparison to sequences from other species (**a**) Percentage of sequence similarity of field pea contigs with nr, nt databases and sequences from other plant species; (**b**) Venn diagram summarising the distribution of BLASTN matches between the Kaspa transcriptome and sequences from three other legume genomes; (**c**) Venn diagram summarising the distribution of BLASTN matches between the Parafield transcriptome and sequences from three other legume genomes. Numbers within the Venn diagram indicate the number of sequences sharing similarity using BLASTN and the numbers within the parenthesis indicate the percentage of matches in terms of total numbers

In order to annotate the transcriptomes, all contigs were BLASTX analysed against the nr database of GenBank. For the Kaspa transcriptome, BLASTX analysis (Additional file 2) revealed 60,808 sequences (47 %) with significant matches, which were then filtered to remove non-plant sequences. This process resulted in a set of 59,229 sequences corresponding to 27,145 unique gene clusters. The length of the annotated sequences varied from 201 to 7,802 bp, with an average of 809 bp, and N50 of 1,106 bp. There were 34,452 (59 %) annotated sequences ≥ 500 bp, in which 15,867 sequences were longer than 1,000 bp, and the remaining 41 % of sequences were 201–500 bp in size. The E-value distribution of significant hits revealed that 48 % of matched sequences exhibited high levels of similarity (E-value lower than 10^−50^) to other legume genomes (Additional file 3, Figure A). For the Parafield transcriptome, 64,727 (43 %) of sequences exhibited significant BLASTX hits (Additional file 2), and after the removal of the non-plant sequences, 63,843 sequences (N50 of 1,083 bp and average 797 bp) remained, corresponding to 27,655 unique genes. Among the annotated sequences, 36,979 (58 %) were greater than 500 bp in length, whereas 26,863 sequences were 201–500 bp in length. The distribution of significant hits for the Parafield contigs showed that 48 % of the sequences displayed E-values less than 10^−50^, while the other matching sequences were located in the value range between 10^−50^ and 10^−10^ (Additional file 3, Figure A). The annotated contigs were also examined for the presence of repetitive elements, and c. 1 % of the contigs were annotated as repeat elements such as retrotransposons, *gag* polyprotein-encoding etc. The distribution of gene annotations based on BLASTX analysis exhibited a highest number of hits against sequences of *M. truncatula*, followed by soybean, and so-far published pea protein sequences within the nr database of NCBI (Additional file 3, Figure B). The BLASTN analysis of transcriptome contigs (Additional file 4) identified a higher number of matches (Fig. 3) to the NCBI nt database as compared to BLASTX analysis against nr. However, most of these additional matches were annotated as retrotransposons and hypothetical proteins, without well-characterised functions. The BLASTN analysis of transcriptome contigs (Additional file 4) against the pea chloroplast genome identified up to 0.17 % of contigs to be chloroplast-derived.

Contigs from the assembly were also BLASTN analysed against both the genomes and CDS of chickpea, *M. truncatula* and soybean. A total of 72,651 (56 %) Kaspa contigs (Additional file 5A and B) and 73,621 (49 %) Parafield contigs (Additional file 5C and D) could be mapped to any of these reference species. Of the total 72,651 Kaspa contigs, 38,924 (53 %) contigs were found to have common matches between chickpea, *M. truncatula* and soybean, while for Parafield out of 73,621 contigs 42,581 (57 %) were found to be common between all reference species. Other contigs were either common between any two of the three references, or specific to each reference (3.5 % to chickpea and *M. truncatula*, 0.7 % to soybean) (Fig. 3).

Reciprocal reference read mapping indicated a large number of contigs that showed matches to the other genotype (87.2 % of Kaspa contigs matched to Parafield reads, and 82.7 % of Parafield contigs matched to Kaspa reads). Among the shared contigs, specific genes known to be essential for plant development and function were identified, including but not limited to chlorophyll a-b binding protein AB80, cytochrome P450, dehydrin-cognate and seed albumin PA1. The contigs with no significant match to the other genotype were also examined and identified as hypothetical proteins, disease resistance genes, stress-related proteins etc.

**Fig. 4:**
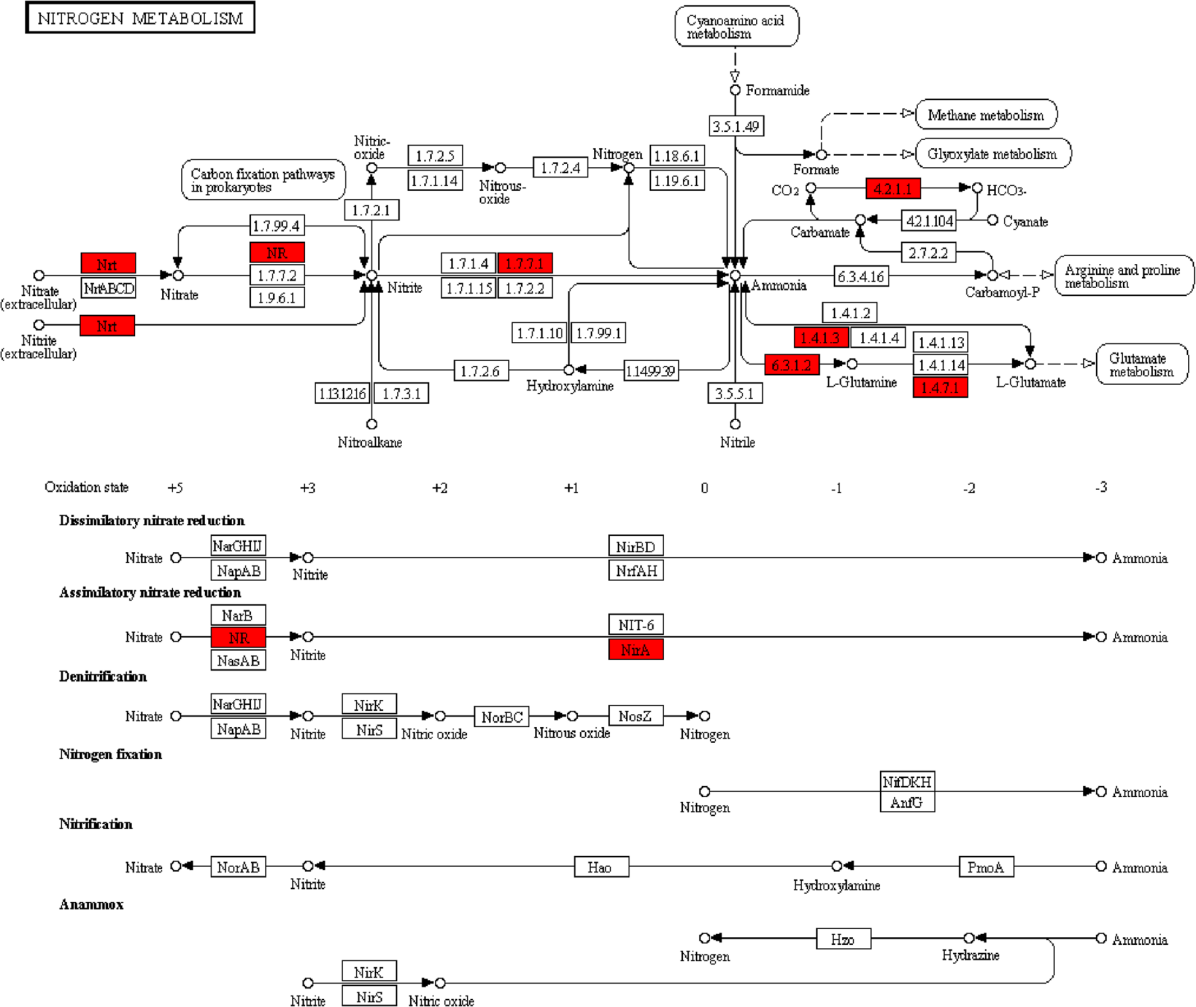
The distribution of field pea contigs against genes encoding enzymes involved in nitrogen metabolism pathways. This is a global nitrogen metabolism pathway map in which a red colour indicates genes identified in data from the present study, all of the known nitrogen metabolism genes in legumes having been identified

In order to characterise the assembled contigs and identify active biological processes, annotated sequences were mapped to the reference biochemical pathways in the KEGG database using eudicot species such as *Arabidopsis thaliana* (L.) Heynh*.*, cocao (*Theobroma cacao* L.), soybean, alpine strawberry (*Fragaria vesca* L.), grapevine (*Vitis vinifera* L.), potato (*Solanum lycopersicum* L.) and rice (*Oryza sativa* sp. *japonica*) as references. In total, 22,056 (37.3 %) contigs from Kaspa and 23,692 (37.1 %) contigs from Parafield were mapped to 157 KEGG pathways corresponding to five modules; metabolism, cellular processes, genetic information processing, environmental information processing and organismal systems (Additional file 6). Metabolic pathways were well represented, most of which were associated with biosynthesis of secondary metabolites, carbohydrate metabolism and amino acid metabolism. Furthermore, mapping of contigs against the glycolysis/gluconeogenesis pathway revealed that all of the genes involved in this pathway were present in the dataset. Another important pathway (nitrogen metabolism), which is crucial to legume species, was also analysed and revealed the presence of all known genes (Fig. 4). In addition, genes for all key enzymes required for the legume-specific isoflavonoid biosynthesis pathway were identified, using *M. truncatula* and chickpea as references.

Comparison of *de novo* assembled contigs from each genotype to previously published pea transcriptome databases indicated that the current activity captured majority of the previously described contigs (99 % of the contigs described by [24], 96 % contigs from [25], 98 % contigs from [23] and 72 % contigs from [40]), representing 33-36 % (46,631 Kaspa contigs and 49,424 Parafield contigs) of the current assemblies (Additional file 7A and B).

**Fig. 5:**
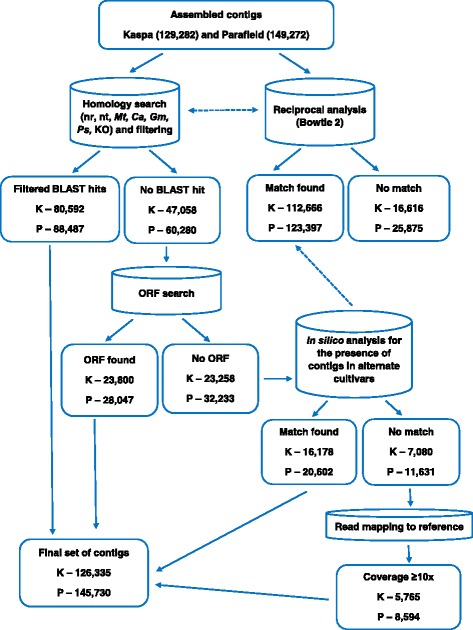
Details of the selection process for field pea contigs. K - Kaspa transcriptome and P - Parafield transcriptome

In summary, a total of 80,592 contigs from Kaspa and 88,487 from Parafield were annotated and characterised using the similarity searches as described. However, a large proportion of contigs from both cultivars (47,058 from Kaspa and 60,280 from Parafield) still remained uncharacterised. These sub-sets were further evaluated and searched for the presence of ORFs. This process identified an additional 23,800 contigs from Kaspa (Additional file 8A) and 28,047 from Parafield (Additional file 8B) which contained a START and STOP codon with minimum sequence length of 100 bp. An additional 16,178 contigs from Kaspa and 20,602 from Parafield were identified in the reciprocal searches (Fig. 5). A final set of contigs (126,335 contigs in Kaspa and 145,730 contigs in Parafield) were compiled after further selection of contigs from the remaining sub-set based on level of coverage (≥10×), although this threshold requirement prevented discovery of lowly expressed novel contigs in field pea. All filtered contigs from both Kaspa and Parafield assemblies were deposited in DDBJ/EMBL/GenBank (accession numbers, Kaspa - GCMF00000000, GCMG00000000, GCMH00000000, GCMI00000000, GCMJ00000000, GCMK00000000 and GCML00000000 and Parafield - GCKA00000000, GCMM00000000, GCMN00000000, GCMO00000000, GCMP00000000 and GCMQ00000000).

### Tissue-specific expression analysis

**Fig. 6:**
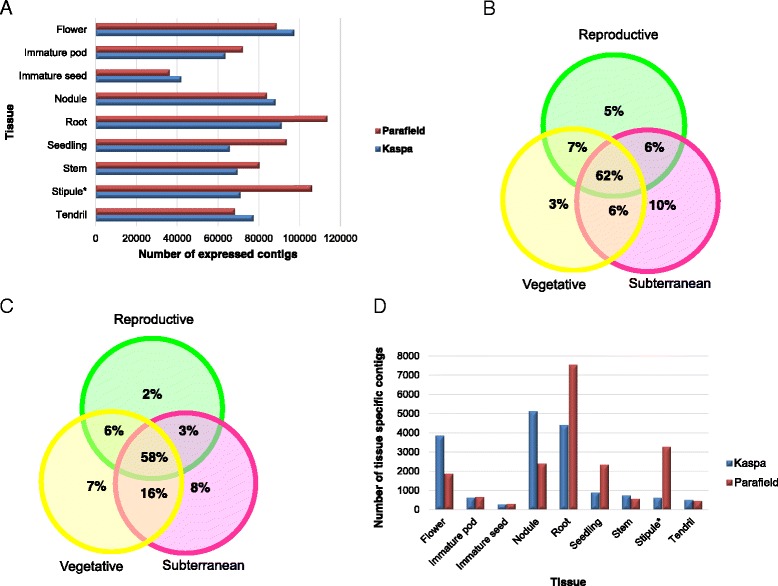
Expression patterns in different tissue samples: (**a**) Number of contigs expressed in each tissue sample; (**b**) Percentage of shared and specific expression profiles of contigs expressed in Kaspa; (**c**) Percentage of shared and specific expression profiles of contigs expressed in Parafield; (**d**) Number of tissue-specific contigs. *For Parafield, stipule and leaflet tissue-derived read counts were merged, while Kaspa contributed only stipule tissue-derived reads

Reads from the individual (tissue-specific) libraries were aligned to genotype-specific assemblies. Most of the tissues showed expression of a similar number of contigs, with the exception of immature seeds for which a relatively lower number was observed (Fig. 6a). Expression of contigs from reproductive tissues, subterranean tissues and vegetative tissues of the two genotypes was compared through use of Venn diagrams (Figs. 6b and c). A total of 62 % of contigs were common between the three groups.

Analysis of tissue-specific expression revealed that roots expressed the maximum number of tissue-specific contigs (Fig. 6d). Flowers and nodules expressed more tissue-specific contigs than immature pods and leaves, while there were very few contigs that were expressed exclusively in immature seed. Detailed contig expression lists for each tissue are provided in Additional file 9A and B. Approximately 87 % of the contigs (109,281 in Kaspa and 130,297 in Parafield) were expressed in more than one tissue in both genotypes. Only a small proportion (0.2 % in immature seed - 5 % in root) of contigs were specific to a particular tissue type (Fig. 6d). Using the read counts obtained from mapping, expression levels of commonly expressed contigs were assessed, revealing that different tissues displayed variable expression levels for the common contigs. For example, storage proteins such as albumin and vicilin were highly expressed in immature seeds compared to other tissues. Similarly, the genes for the small sub-unit of ribulose bisphosphate carboxylase-oxygenase (RUBISCO) and the light-harvesting chlorophyll-a/b binding (CAB) protein Lhcb1 were expressed at a much higher level in leaves when compared to other tissues. Assessment of the annotation of tissue-specific contigs indicated predominant involvement in functions particular to that tissue type (Additional file 10).

A high level of expression was noted from root, root-tip and nodule tissues from read mapping. Further analysis of contigs from these tissues to fungal and bacterial sequences revealed that only a small percentage of reads showed matches to non-plant references, of which *Rhizobium* was one of commonly represented species, particularly (as expected) in nodule-derived assemblies (0.5 % of nodule, 0.05 % of root-tip and 0.01 % of root mapped reads from Kaspa exhibited similarity to *Rhizobium*, as compared to 0.3 % of nodule and 0.01 % of root tissues mapped reads from Parafield).

## Discussion

### De novo *sequence assembly and functional annotation of the field pea transcriptome*

Legume species such as peas are economically important as sources of food for humans, feed for livestock and contributors to sustainable agriculture due to the ability to fix atmospheric nitrogen in symbiotic association with *Rhizobium* bacteria, hence providing crop plants with a free and renewable source of nitrogen [42]. A fundamental understanding of the field pea transcriptome will provide an overview of the genes, regulatory networks and functional roles that govern these key biological processes [43]. Additional genomic and transcriptomic resources may permit discovery of novel genes associated with multiple agronomic traits useful for plant breeding purposes.

The RNA-Seq approach has a wide range of applications, including investigation of different biological processes at the tissue or cell level [44], description of the entire transcriptome of a given organism [17], and assessment of genetic diversity on an evolutionary time scale [45]. RNA-Seq technology has previously been used to characterise the transcriptomes of a number of plant species, including maize and soybean [15, 16, 18], and decreasing costs of DNA sequencing technologies will provide opportunities for the generation of equivalent information for many more species in future. Due to developments in sequence analysis software, transcriptome studies are now possible for species that do not as yet have a reference genome [46, 47], of which field pea is an example.

In order to characterise the field pea transcriptome, two commonly cultivated Australian field pea cultivars (Kaspa and Parafield) were subjected to sequencing of cDNA samples. A range of different tissues were included in order to obtain effective sampling of transcript complexity and to maximise the probability of detecting mRNA of low abundance. In total, c. 408 million and 352 million high quality reads were used for *de novo* assembly, generating 129,282 and 149,272 contigs for Kaspa and Parafield respectively. The number of contigs generated in the current study is comparable to that from other studies [17], which used Illumina sequencing data and Trinity assembler. Moreover, SOAP-denovo-Trans assembler also generated a similar number of contigs, with a lower N50 value and therefore not used for further analysis.

Approximately 50 % of the contigs were annotated by comparison with the NCBI nr database. The majority of contigs exhibited significant matches to *M. truncatula* sequences, followed by those from soybean. However, only 3.3 % of the BLASTX-mediated matches were to pea-derived protein sequences, probably due to the limited number of proteins currently available in the NCBI database (3,689), as opposed to failure to recognise homologous sequences. As a consequence, the number of observed matches reflects not only the degree of relatedness between comparator species, but also the scope of available sequence data. Nonetheless, the proportion of similarity of field pea transcriptome sequences against the NCBI nr database is comparable to results from other species, such as sweet potato (*Ipomoea batata*) [44].

The field pea transcriptome assembly contained small sequences as well as unigenes containing more than one sequence. Trinity assembler generates high numbers of putative transcripts, including alternatively spliced isoforms and transcripts from recently duplicated genes which lead to the generation of similar transcripts [48]. Moreover, Trinity-derived transcripts are not scaffolded across sequencing gaps, which may also lead to generation of a large number of small transcripts. The small-sized sequences (in the range 200–300 bp) may be too small for BLASTX analysis, and may hence have failed to detect similarity to any known proteins [49]. Alternatively, the small sequences may encode novel proteins, or be derived from untranslated regions (UTRs) or non-coding RNAs (ncRNAs) [17]. The multiple contigs that were assembled into unigene clusters may represent transcription variants, allelic variants, closely related paralogous sequences, misassembled transcripts, or transcripts that were fragmented due to low coverage [46].

Sequence similarity of field pea contigs to the genomes and transcriptomes of other legume species was determined using BLASTN analysis, revealing levels of conservation up to 56 %. Comparable results were observed for the chickpea transcriptome in similarity searches against other legumes [6]. Moreover, up to 57 % of the annotated contigs were common to all of the legume genomes used in the present study. BLAST analysis revealed a highest level of similarity to sequences from chickpea, followed by *M. truncatula*, and more distantly, by soybean. Field pea, chickpea and *M. truncatula* all belong to the Galegoid clade of cool-season legumes, and are hence mutually more closely related than to soybean, which belongs to the Phaseoloid clade of warm-season legume species within the Papilionoideae [50]. A comparatively lower level of similarity to soybean genomic sequences, is hence not unexpected, considering the evolutionary divergence of the various species. However, the rank order of similarity within the Galegoid clade is not so easy to rationalise, as the Vicieae and Trifolieae tribes (to which field pea and *M. truncatula* belong) are thought to share a common ancestor more recently in evolutionary time than with the Cicereae tribe, to which chickpea belongs [51]. A proportion of the unmatched field pea contigs may genuinely represent species-specific components of the field pea transcriptome. Similarity searches against the genomes of the legume comparators identified matches to field pea contigs which did not display matches to the CDS, possibly due to incomplete annotation of those genomes. Previous studies have also reported that clusters of reads from transcriptome sequencing were mapped to the unannotated regions of the genome [52].

Within the KEGG analysis, well-represented pathways in the field pea transcriptome included those involved in carbohydrate metabolism, biosynthesis of secondary metabolites, amino acid metabolism, lipid metabolism and energy metabolism. All of the expected genes involved in the isoflavonoid biosynthesis pathway were identified, though expression was lower than that of the genes involved in synthesis of other secondary metabolites such as phenylpropanoids and flavonoids. In addition, activities associated with genetic information processing (spliceosome, ribosome and RNA transport functions), plant-pathogen interactions and plant hormone signal transduction were identified.

The two field pea genotypes used in this study differ in terms of morphology and resistance to different abiotic and biotic stresses. Kaspa is a high-yielding, late flowering field pea variety with excellent pod-shatter resistance, good lodging tolerance, resistance to downy mildew and improved resistance to black spot. The Parafield cultivar is mid- to late-season flowering, with moderate resistance to pod-shattering, moderate resistance to bacterial blight and tolerance to saline soil toxicity. The observed differences in gene expression between genotypes may account for some of the performance differences between cultivars. Reciprocal sequence analysis identified 23-27 % of contigs which displayed no significant match to any transcript in the other genotype. Those contigs may not be present in the other genome, or much more likely, have not been expressed at a sufficiently high level to undergo assembly. Based on sequence annotation, the major differences in gene expression between the two genotypes were identified as being associated with abiotic/biotic stress tolerance (at low levels), transcription factors and signal transducers. Conversely, the genes held in common between the two genotypes included those encoding proteins known to be necessary for development and function, such as chlorophyll a-b binding protein AB80, dehydrin-cognate, cytochrome P450, disease resistance proteins and ABC transporters.

For both the Kaspa and Parafield transcriptomes, the proportion of sequences that are present in previous pea transcriptome datasets [23–25, 41] were assessed, revealing that from 72 to 99 % of the unigene sets from those assemblies were regenerated in the current study. Based on this comparisons, it would appear that the current study was able to reconstruct a higher number of assembled contigs than those obtained from other assembly processes.

After step-by-step annotation and classification of the field pea transcriptome, totals of 126,335 contigs in Kaspa and 145,730 contigs in Parafield were obtained, representing 71,014,518 bp and 79,440,852 bp of cumulative sequence, respectively. These sets include contigs that may represent alternatively spliced forms of the same gene locus. Although this sampling process has been highly effective, a determination of the exact composition of the pea transcriptome will require a corresponding genomic sequence assembly, permitting annotation and classification of a broader range of transcripts. As c. 40 % of the sequences generated in the present study lacked significant similarity to genes of known function, alternative computational means were used to identify more sequences, such as identification of ORFs, use of reciprocal analysis and relative coverage in the transcriptome. The results of these analyses were used to annotate and classify the remaining sequences, but these processes are still be prone to exclude contigs with low levels of expression. Limited sequencing depth of lowly expressed contigs can cause sequencing biases, resulting in the partial assembly of contigs which may fail to be classified by comparison to known gene annotations. A high proportion of these lowly expressed contigs may be derived from pea-specific repetitive elements, belonging to several sub-families which are highly variable in sequence and hence individually present in relatively lower copy number [2].

### Tissue-specific expression analysis

In order to identify and characterise expression of contigs on a tissue-specific basis, reads from different libraries were aligned to the genotype-specific assemblies. The number of contigs detected was similar for most samples, with the exception of those from seeds, despite generation of a similar number of reads. A similar observation was reported in a previous study of seed-specific transcription in *A. thaliana* [10]. Despite the similar number of active contigs in each sample, expression dynamics varied considerably between tissues, the largest number of contigs showing preferential expression in root. Substantial overlap in expressed contigs was identified between reproductive, vegetative and subterranean tissue-derived clusters, c. 62 % of contigs being attributed to a generic expression profile, while smaller cohorts displayed tissue-specific expression. The root tissue-derived group displayed the most diverse transcriptome, as compared to the reproductive tissue-derived group which may be associated with regulation of root apical meristem cells, pathogen resistance, symbiosis and immune responses [53, 54]. A larger number of vegetative tissue-specific contigs were identified in the Parafield transcriptome as compared to that from Kaspa, possibly because Parafield contributed sample from leaf tissue, in addition to stipule-derived contigs which were common to both.

The analysis was performed in more detail at the level of individual organ types, which demonstrated that roots contributed the largest number of tissue-specific contigs, followed by flower and nodule, while immature seed contributed the least. The identity of organ types that contribute the largest number of tissue-specific contigs varies between legume species, from nodules and flowers in *L. japonicus* [14] and soybean [55], to flower bud and immature pod in chickpea [56]. Assessment of the annotation of some of the contigs that are expressed only in nodule tissue (such as those for nodule inception protein and nodulin) revealed involvement in nodule developmental processes [57, 58]. Likewise, some of the contigs expressed specifically in roots (such as those for hyoscyamine 6-dioxygenase, acyltransferase, MtN19 protein and germin-like protein) were associated with root development, root wax formation, and defence/wounding-related process that are also implicated in the legume-Rhizobium symbiosis [53, 54]. The MADS box protein PIM, which is represented only among flower contigs regulates floral meristem identity in pea [59]. Many immature seed-specific contigs represented transcription factors, including the BZIP transcription factor that is involved in seed maturation [60]. The results of tissue-specific analysis indicate that different tissues express distinct contigs, many of which are clearly related to biological functions, providing a unique transcriptome signature for that tissue. These tissue-specific contigs may provide further insight into specialised organ-specific biochemical, physiological, and developmental processes.

The contigs that were expressed at very low levels without annotation and classification, and also without any reciprocal match to the transcriptome of the other cultivar were further analysed. The lowly expressed cultivar-specific contigs were preferentially associated with nodule and root tissues in both transcriptomes. Field pea root and nodule tissues may hence possess novel contigs associated with specific functions such as rhizobial symbiosis and nitrogen fixation, or these contigs could represent sequences from novel bacteria, although additional studies will be required for validation of this hypothesis.

## Conclusions

The present study has demonstrated that RNA-Seq technology provides an efficient method for transcriptome analysis of non-model plant organisms, delivering a valuable resource of gene expression data for further analysis. Gene annotation and understanding of potential pathways provides the basis for investigation of specific processes, biological functions, gene interactions and mechanisms involved in different agronomic traits. The transcript expression patterns were generally similar between different tissues, but the tissue-specific contigs from different libraries displayed signatures which were consistent with biological expectations. The combined transcriptomes of two contrasted varieties provide a key resource for identification of DNA sequence variants for use in genomics-assisted breeding of field pea. In conclusion, the present study has substantially increased the transcriptome resources that are available for use in varietal improvement of this important grain legume species.

### Availability of supporting data

The data sets supporting the results of this article are included within the article and its additional files. The sequence data has been deposited at DDBJ/EMBL/GenBank under the SRA accessions - SRR1910794, SRR1910804-SRR1910826, SRR1913075, SRR1913256 and SRR1913731-SRR1913750. Transcriptome Shotgun Assembly project has been deposited at DDBJ/EMBL/GenBank under the accessions, Kaspa - GCMF00000000, GCMG00000000, GCMH00000000, GCMI00000000, GCMJ00000000, GCMK00000000 and GCML00000000 and Parafield - GCKA00000000, GCMM00000000, GCMN00000000, GCMO00000000, GCMP00000000 and GCMQ00000000. The version described in this paper is the first version. BioProject ID for Kaspa transcriptome is PRJNA277074 and for Parafield transcriptome is PRJNA277076.

## Additional files

Additional file 1:
**Summary of sequencing outputs.** This file contains a summary of the sequencing outcomes for each tissue-specific library of both varieties, Kaspa and Parafield (total number of raw reads from both sequencing platforms, number of reads used for assembly). (XLSX 11 kb) Additional file 2:
**Bioinformatic annotation (BLAST) of Kaspa and Parafield contigs against the nr database.** The information includes the BLASTX results obtained as a result of comparison of Kaspa and Parafield contigs against the nr database at a threshold E value < 10^−10^. (XLSX 10037 kb) Additional file 3:
**BLASTX alignment details.** (A) This figure shows the E-value distribution of significant hits for Kaspa and Parafield transcriptomes; (B) This figure represents the BLASTX top-hit species distribution of contig annotations. (PDF 34 kb) Additional file 4:
**Bioinformatic annotation (BLASTN) of Kaspa and Parafield contigs against the nt database and the pea chloroplast genome.** The information includes the BLASTN results obtained as a result of comparison of Kaspa and Parafield contigs against the nt database and the pea chloroplast genome at a threshold E value < 10^−10^. (XLSX 10124 kb) Additional file 5:
**(A) Bioinformatic annotation (BLASTN) of Kaspa contigs against the chickpea**, ***M. truncatula*** **and soybean CDS.** The information includes the BLASTN results obtained as a result of comparison of Kaspa contigs against the chickpea, *M. truncatula* and soybean CDSs at a threshold E value < 10^−10^. (B) Bioinformatic annotation (BLASTN) of Kaspa contigs against the chickpea, *M. truncatula* and soybean genome: The information includes the BLASTN results obtained as a result of comparison of Kaspa contigs against the chickpea, *M. truncatula* and soybean genomes at a threshold E value < 10^−10^. (C) Bioinformatic annotation (BLASTN) of Parafield contigs against the chickpea, *M. truncatula* and soybean CDS: The information includes the BLASTN results obtained as a result of comparison of Parafield contigs against the chickpea, *M. truncatula* and soybean CDSs at a threshold E value < 10^−10^. (D) Bioinformatic annotation (BLASTN) of Parafield contigs against the chickpea, *M. truncatula* and soybean genome: The information includes the BLASTN results obtained as a result of comparison of Parafield contigs against the chickpea, *M. truncatula* and soybean genomes at a threshold E value < 10^−10^. (ZIP 52127 kb) Additional file 6:
**Pathway assignment based on KEGG.** This file summarises the properties of Kaspa and Parafield contigs annotated to the reference pathways in KEGG database, and the contigs with KO annotations. (XLSX 915 kb) Additional file 7:
**(A). Bioinformatic comparison (BLASTN) of Kaspa contigs against the pea transcriptome datasets.** The information includes the BLASTN results obtained as a result of comparison of Kaspa contigs against four other transcriptome datasets of pea at a threshold E value < 10^−10^. (B) Bioinformatic comparison (BLASTN) of Parafield contigs against the pea transcriptome datasets: The information includes the BLASTN results obtained as a result of comparison of Parafield contigs against four other transcriptome datasets of pea at a threshold E value < 10^−10^. (ZIP 31071 kb) Additional file 8:
**ORF prediction of the unannotated contigs.** (A) This file contains information on ORFs prediction for the unannotated Kaspa-derived contigs. (B) This file contains information on ORFs prediction for the unannotated Parafield-derived contigs. (ZIP 7244 kb) Additional file 9:
**Transcript expression for each tissue.** (A) This file details contigs expression for the individual (tissue-specific) libraries after alignment to the Kaspa assembly. (B) This file details contigs expression for the individual (tissue-specific) libraries after alignment to the Parafield assembly. (ZIP 20813 kb) Additional file 10:
**Annotation of tissue-specific contigs.** This file contains information on the annotation of some of the tissue-specific contigs. (XLSX 10 kb)

## Abbreviations

ABC transporters: ATP-binding cassette transporters
bp: Base pair
BLAST: Basic Local Alignment Search Tool
BZIP: Basic leucine zipper
CAB: Chlorophyll-a/b binding
cDNA: Complementary DNA
CDS: Coding DNA sequences
DNA: Deoxyribonucleic acid
Gb: Giga base pair
KEGG: Kyoto Encyclopedia of Genes and Genomes
Mb: Mega base pair
mRNA: messenger RNA
NCBI: National Center for Biotechnology Information
ncRNAs: non-coding RNAs
ORF: Open reading frame
PVP: Polyvinylpyrrolidone
RNA: Ribonucleic acid
RNA-Seq: RNA sequencing technology
RUBISCO: Ribulose bisphosphate carboxylase-oxygenase
UTRs: Untranslated regions
W/V: Weight/volume
