# Supplementary figures and images for: De novo assembly and characterisation of the field pea transcriptome using RNA-Seq
Source: BMC Genomics. 2015 Aug 16;16(1):611. doi: 10.1186/s12864-015-1815-7 (PMC4537571; doi:10.1186/s12864-015-1815-7)

A

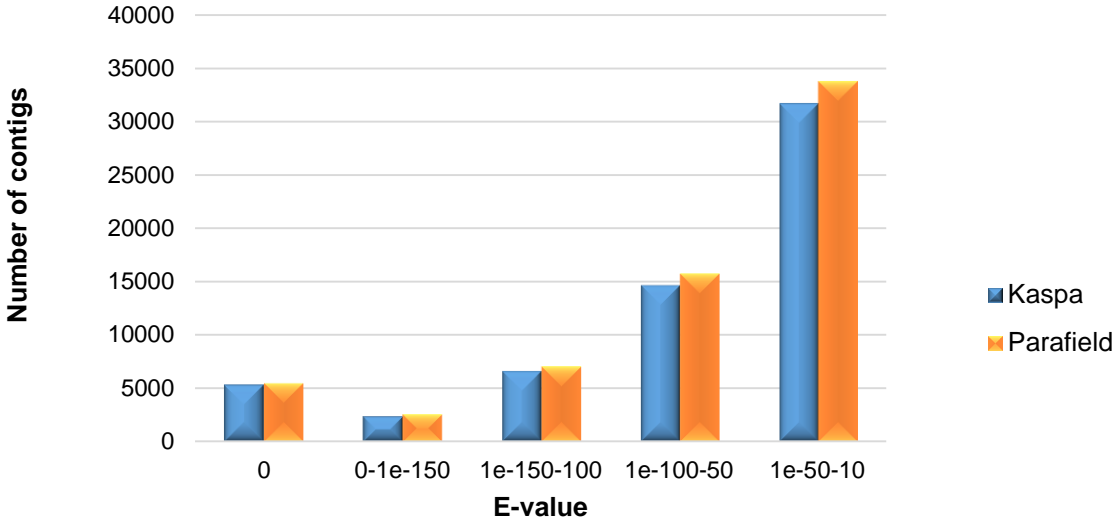

B

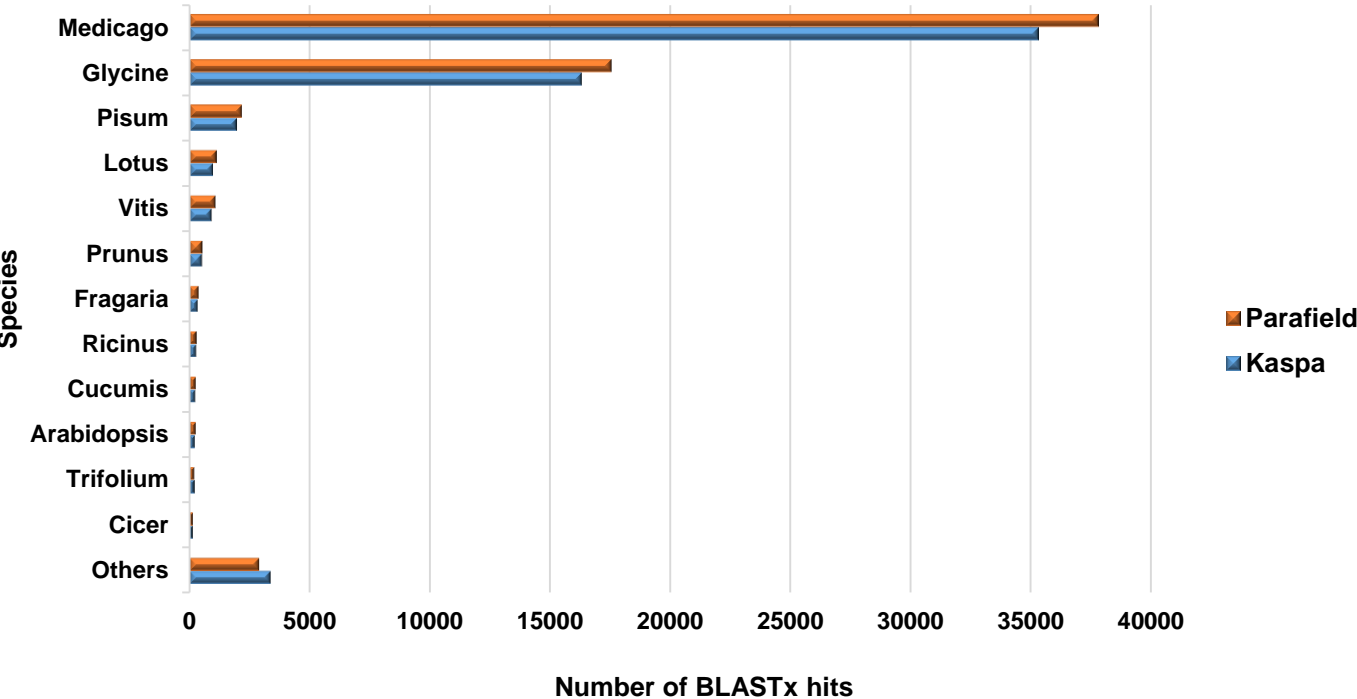

Supplement: Additional file 3: — BLASTX alignment details. (A) This figure shows the E-value distribution of significant hits for Kaspa and Parafield transcriptomes; (B) This figure represents the BLASTX top-hit species distribution of contig annotations. (PDF 34 kb) [file 12864_2015_1815_MOESM3_ESM.pdf]
